# The clinical characteristics of benign oral mucosal tumors

**DOI:** 10.4317/medoral.19387

**Published:** 2013-12-07

**Authors:** Irit Allon, Ilana Kaplan, Gavriel Gal, Gavriel Chaushu, Dror M. Allon

**Affiliations:** 1 Department of Oral Pathology & Oral Medicine, Goldschleger School of Dental Medicine, Tel-Aviv University, Israel; 2 Institute of Pathology, Rabin Medical Center, Petah-Tikva, and Sackler School of Medicine, Tel-Aviv University, Israel; 3 Departments of Oral and Maxillofacial Surgery, Rabin Medical Center, Petah-Tikva, and of Oral Surgery, Goldschleger School of Dental Medicine, Tel-Aviv University, Israel

## Abstract

Objectives: To investigate the clinical characteristics and pre-biopsy provisional diagnoses of benign oral mucosal tumors. Material and Methods: A 10- year retrospective analysis of all benign tumors of the oral mucosa, from a university- affiliated oral and maxillofacial surgery department. Results: 146 benign tumors were included. The mean age was 49.6 years, with an approximately equal gender distribution. The most prevalent tumor types were lipomatous tumors (27.4%), vascular (23.3%), and salivary gland tumors (16.5%). Tongue, labial and buccal mucosa were the most frequently involved sites. The vast majority (98.6%) presented as non-ulcerated masses. Only 2 (1.4%) presented as ulcerated masses. The clinical provisional diagnosis correctly classified lesions as non-malignant in 93.3%. In only 9 (6.7%) suspicion of malignancy was included in the provisional diagnosis. However, benign neoplasia was unsuspected in 42.1% of tumors. These cases were clinically classified as reactive. Conclusions: Benign tumors were most likely to be clinically correctly classified as non-malignant, but even in the setting of experienced oral surgeons, neoplasia was unsuspected in more than 40% of cases. This data strongly supports the need to biopsy every oral mucosal mass, since inaccurate clinical evaluation of the lesion’s biological nature was a frequent event.

** Key words:**Malignant, benign, reactive, ulcerated mass, non-ulcerated mass, clinical diagnosis.

## Introduction

The diagnosis of a wide range of lesions occurring in the oral mucosa is a vital part of dental practice. One of the main tools in developing a list of potential diagnosis for a lesion is knowledge of the frequency of each potential lesion type (1). This information can provide clinicians with the data to predict the probability of its occurrence. Unfortunately, information from the literature relating to the frequency of oral benign mucosal tumors is uncommon. Studies relating to benign tumors of the oral mucosa have been published in the literature, focusing on clinico-pathologic correlations of lipoma variants (2-5), salivary gland tumors (6-9), hemangiomas (10-12), neural (13-14) and other soft tissue mucosal tumors (15-21). Most of these do not relate directly to the clinical appearance and rate of ulceration of the tumors but rather, describe oral mucosal benign tumors as swellings or raised lesions. None of these studies investigated the accuracy of the pre- biopsy clinical in comparison to the final diagnosis. In text books and large scale screening studies benign tumors are described as raised masses or swellings (22-25), but a comprehensive analysis focusing on the spectrum of the benign oral mucosal tumors, including the clinical appearance and accuracy of the clinical differential diagnosis has not been found.

In a recently published 10-year retrospective analysis of malignant tumors of the oral mucosa, we found that close to 60% of oral malignancies presented as non-ulcerated masses, 20% presented as ulcerated masses and 12% as indurated ulcers (26). This pointed out that within the study sample, non-ulcerated masses rather than indurate ulcers were the most common clinical presentations of oral mucosalmalignancies. Another unexpected finding was that approximately one third of the oral mucosal malignancies were not suspected to be malignant prior to biopsy.

The aims of this study were to investigate the clinical characteristics and pre- biopsy provisional diagnoses of benign tumors of the oral mucosa.

## Material and Methods

The study was conducted as a 10- year retrospective analysis. For the present study the archives of the Institute of Pathology of the Rabin Medical Center, Patah-Tikva, Israel were screened for benign tumors of the oral mucosa. The study included only diagnostic biopsies which were microscopically diagnosed as benign tumors, while those lacking information on clinical presentation were excluded. The pre-biopsy clinical differential diagnoses with which the biopsies were submitted were classified into reactive/ developmental lesions, benign tumors or malignancy, as previously described (27). In cases that included more than a single provisional diagnosis, the analysis included the classification which was the most severe possibility. Therefore, in cases that included a request to rule out a malignancy, the clinical diagnosis included in the analysis was of malignancy. Being a university-affiliated institution training residents for national boards in oral and maxillofacial surgery, high clinical standards are implemented for clinical examination and reporting in patients’ charts. All biopsies were performed by senior members of the department (GG, GC, DMA) with 9-35 years of experience, or by residents closely supervised by them. The study was approved by the institutional review board.

## Results

**Figure 1:**
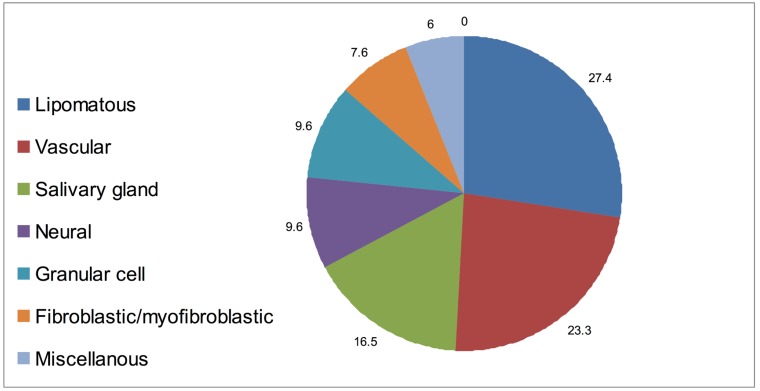
The incidence of benign tumor groups in the oral mucosa.

During a 10-year period (2001-2011), a total of 146 benign tumors of the oral mucosa met the inclusion criteria (Fig. 1). There were 78 males and 68 females, representing an approximately equal gender distribution ( Table 1).

**Table 1:**
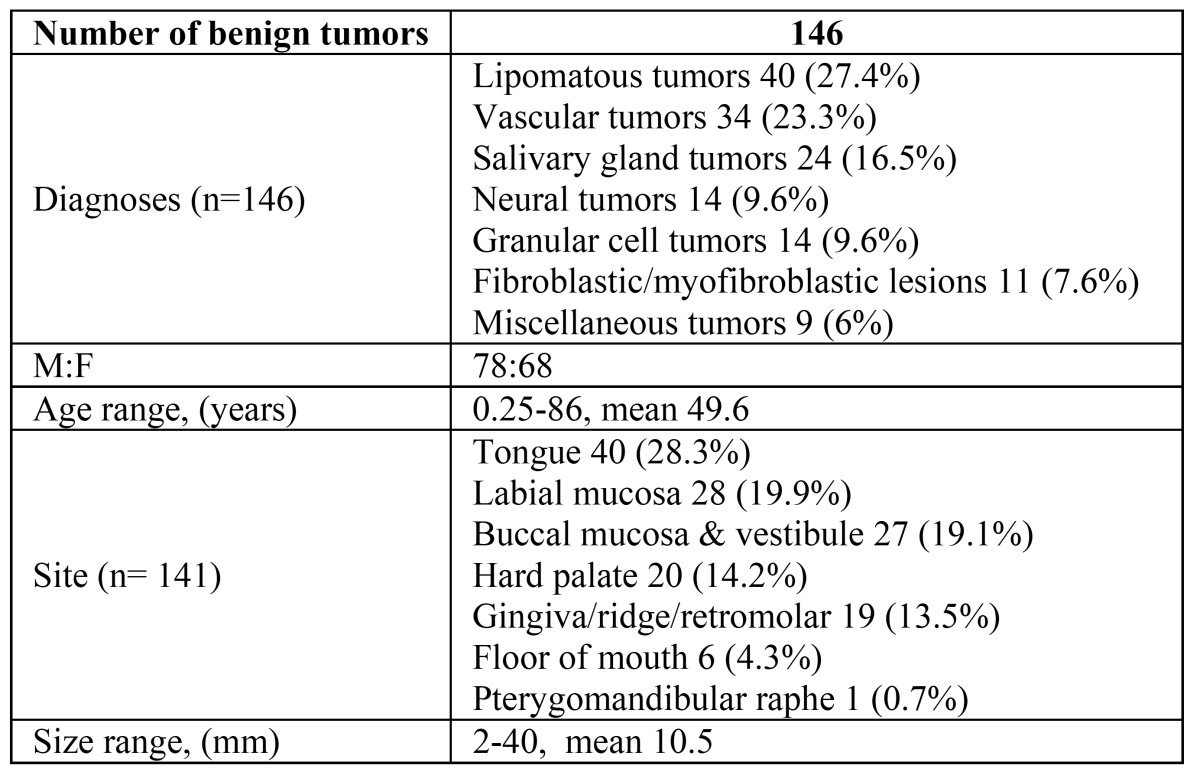
Benign tumors of the oral mucosa, clinical data.

**Figure 2:**
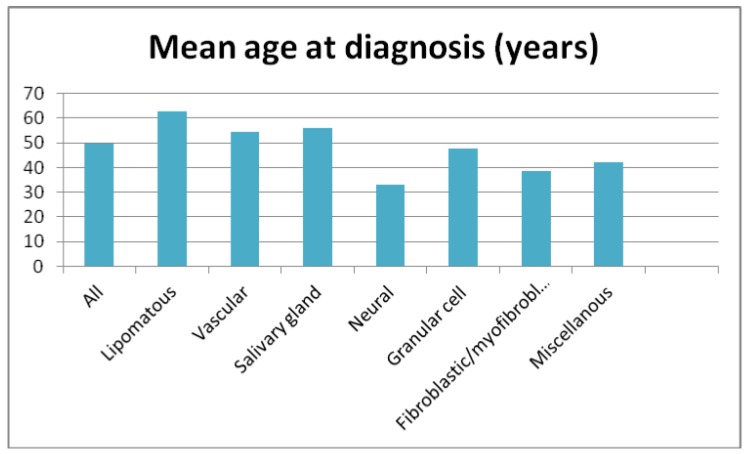
Mean age at diagnosis of benign tumors in the oral mucosa.

The most prevalent tumor types were lipomatous tumors (27.4%), hemangioma variants (23.3%), and salivary gland tumors (16.5%). Other groups included neural and granular cell tumors (9.6% each), fibroblastic/ myofibroblastic (7.6%), with the remaining classified as miscellaneous ( Table 2). The age range was wide, (3 months-86 years) with a mean age of 49.6 years. The mean age at diagnosis of the lipomatous and salivary gland tumors was higher than that of the neural and fibro-blastic/myofibroblastic tumors (over 50 and below 40 years, respectively) (Fig. 2).

**Table 2:**
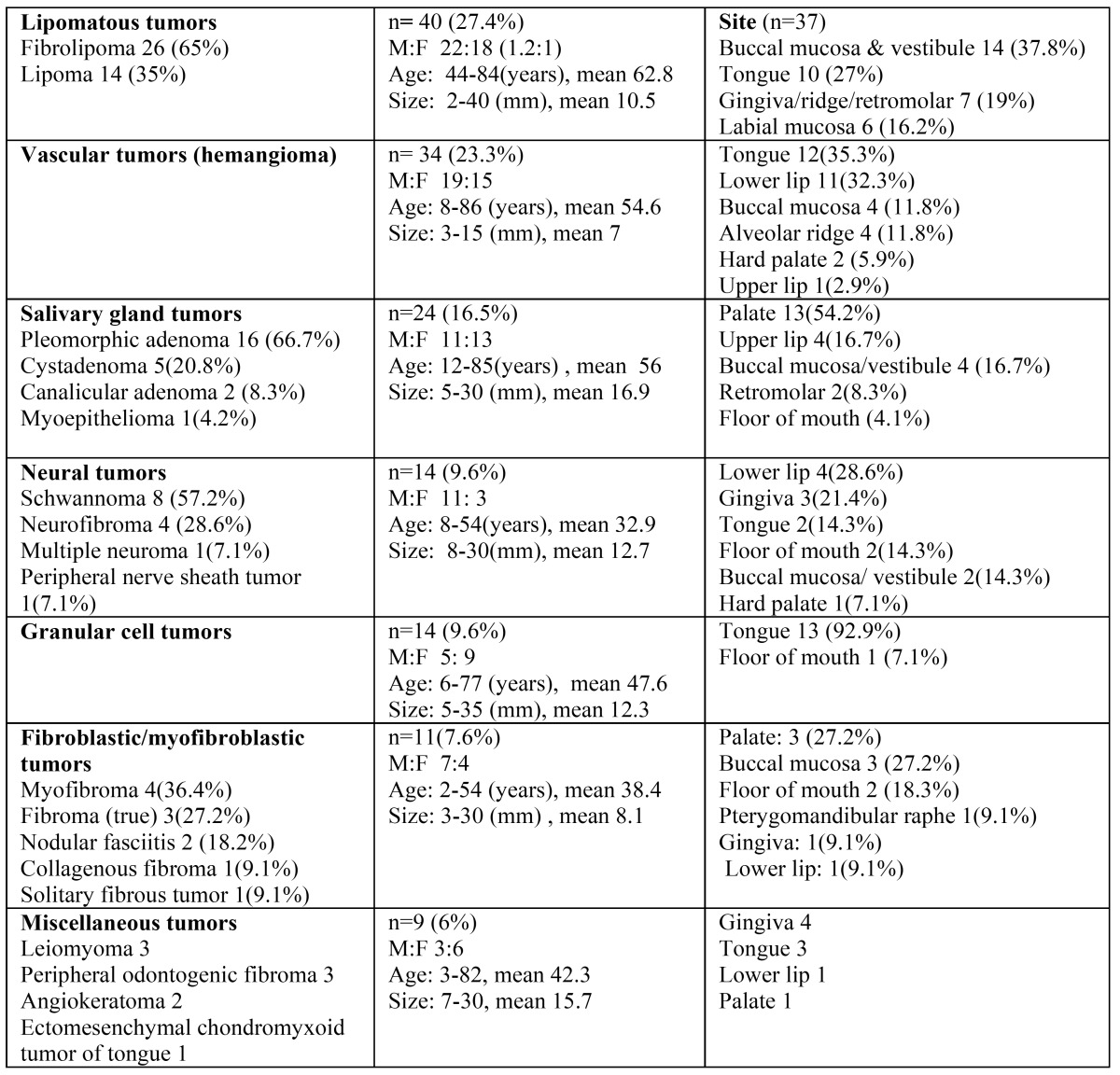
Clinical characteristics of the tumor groups involving oral mucosa.

The mobile tongue was the most frequently affected site (28.3%), followed by the labial mucosa (19.9%), buccal muco-sa/vestibule (19.1%), hard palate (14.2%), gingiva/alveolar ridge (13.5%) and floor of mouth (4.3%). The lesion’s size ranged between 2-40 mm, mean 10.5.

The vast majority (98.6%) of the benign tumors in the oral mucosa presented as a non-ulcerated masses ( Table 3). Only 2 cases (1.4%) presented as ulcerated masses. These included one cavernous hemangioma of the lower lip in a 30 years old male and a granular cell tumor of the tongue in a 6 years old boy. The clinical description of ulceration was validated microscopically in both cases, and none of the cases in which ulceration had not been clinically described showed microscopic ulceration.

**Table 3:**
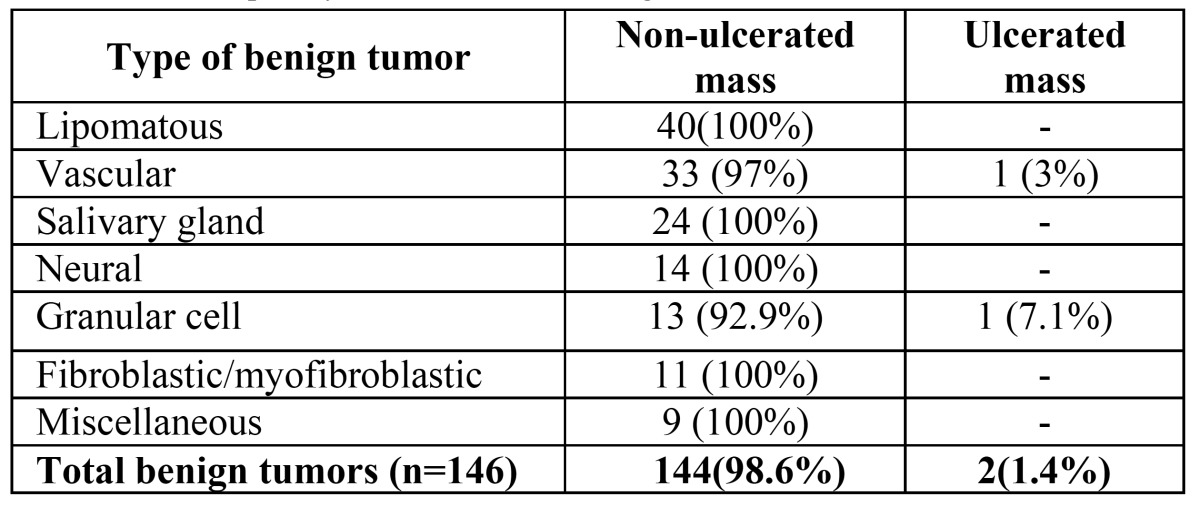
The frequency of ulceration in benign tumors.

In regard to the pre-biopsy provisional diagnoses, 126 (93.3%) were correctly classified clinically as non-malignant, while only in 9 (6.7%) benign tumors arose suspicion for malignancy, (6.7% false negative) ( Table 4). In 73 (57.9%) cases, the provisional diagnoses of a benign tumor was correct, while in 53(42.1%) benign neoplasia was an unsuspected finding, these cases were considered reactive lesions in the provisional diagnosis.

**Table 4:**
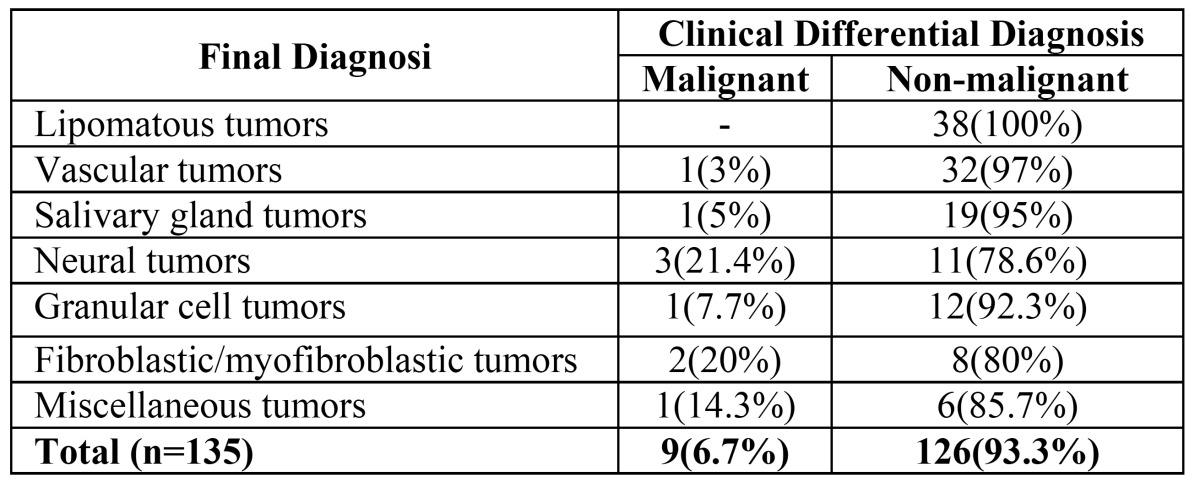
The accuracy of provisional clinical diagnosis, classification as benign or malignant.

## Discussion

The objective of the present study was to provide a comprehensive view of the spectrum of benign tumors of the oral mucosa. Lipomatous, vascular (hemangioma) and salivary gland tumors, in descending order, were the most prevalent in this series. The tongue, labial and buccal mucosa were the most prevalent sites of presentation.

There seemed to be differences in the age distribution between tumor groups: while neural tumors and fibroblastic/myofibroblastic tumors had a mean age under 40 years, lipomatous, vascular and salivary gland tumors presented a mean age between 54-63 years. However, there was a wide range with considerable overlap between the groups.

The size of benign tumors ranged between 2-40 mm in diameter for the entire study group. The salivary gland tumors presented the highest mean size (16.7 mm), while the vascular and fibroblastic/myofibroblastic tumors presented the lowest mean size (7 and 8.1 mm respectively).

Almost all the benign tumors included in the study presented as non- ulcerated masses. Only two presented as ulcerated masses and none as indurate ulcers or flat lesions. Most of the benign tumors were clinically classified correctly as non- malignant and did not raise any clinical suspicion for malignancy. However, more than forty percent of these benign tumors were clinically thought to be reactive, and did not raise suspicion of neoplasia. The results of the present series emphasize that the ulceration rate of benign oral mucosal tumors is very low, a feature that up till the present was described only in sporadic reports such as an ulcerated canalicular adenoma from the palate (8-9) or an ulcerated myofibroblastic tumor (16).

This study follows a previous study on malignancies of the oral mucosa (26). When comparing the results of the present study to those from the malignant oral mucosal tumors, performed in the same center and same time period, it is evident that while in the benign tumors 98% presented as non-ulcerated masses, in the malignant group there was a significantly higher fraction presenting as ulcerated masses (20.4%) or indurate ulcers (11.9%), although still more than half (59.7%) of the malignancies were non- ulcerated at presentation (26). These differences were statistically significant (*p*<0.001, chi-square).

When considering the pre-biopsy classification as malignant or non-malignant lesions (reactive, developmental or benign tumors), the accuracy was significantly higher in the benign tumor group (93.3%), compared with only 68.9% in the malignant tumor group. This implies a significantly higher fraction of false negatives in clinical identification of malignancies (31.1% in the malignant compared to 6.7% in benign groups, *p*<0.001, chi-square). Interestingly, the percent of unsuspected neoplasia in the benign tumors (42.1%), was quite similar to the percent of unsuspected malignancy in the malignant tumor group (31.1%). The data presented was obtained from a medical teaching center and even in this context of highly skilled clinicians, 31.1-42.1% of both malignant and benign neoplasia where unsuspected as such prior to biopsy. This highlights the fact that the ability to recognize a neoplasm on a clinical basis is quite limited. Since non- ulcerated masses are the typical presentation of almost all benign mucosal tumors (present results), more than half of the malignant tumors (26) and the majority of reactive lesions (27), the ability to predict the true biological nature of oral mucosal masses using visual inspection alone is relatively poor, thus, diagnostic biopsy stays an essential and mandatory tool for diagnosis of all masses of oral mucosa. Although the majority of mucosal masses would turn out to be reactive following microscopic analysis, they must not be ignored since both benign and malignant neoplasms cannot be differentiated clinically from reactive lesions with a high degree of confidence. In the decision whether a mass should be removed and submitted for analysis, or followed-up, the allusive “clinical judgment” should play a minimal role. There should be stronger emphasis on the need to biopsy every oral mass, regardless of its ulceration status.

A few possible explanations to the different rate of ulceration between benign and malignant tumors may be proposed: size-related, site- related and biological aspects. Regarding the size, it is possible that as lesions grow they interfere with anatomical structures, compromise the blood supply, or may become traumatized during functional movements such as mastication and speech and therefore ulcerate. However, there was a significant overlap in the size range between the benign and malignant groups (2-40 mm versus 5-30 mm respectively), therefore, size alone would not be a contributing factor for ulceration.

Regarding the site, it is possible that lesions appearing in specific sites may be easily traumatized. However, the tongue was the most common site for both malignant (26) and benign tumors (present study), so it appears that the site distribution cannot offer an adequate explanation for the difference in the clinical presentation. Therefore, additional contributing factors related to the different biological characteristics of the tumors may possibly account for the differences. Within the group of malignancies, there were also differences between the tumor types in the tendency to become ulcerated: SCC ulcerated more frequently than other tumor types, supporting the assumption that differences probably result from variations in biological characteristics (26).

In conclusion, the clinical ability to recognize benign mucosal neoplasms by visual inspection is relatively poor. In about 40% of the benign mucosal tumors, neoplasia was clinically unsuspected, thus, the need to biopsy every oral mucosal mass is supported.
